# Emergency Department Pediatric Readiness and Disparities in Mortality Based on Race and Ethnicity

**DOI:** 10.1001/jamanetworkopen.2023.32160

**Published:** 2023-09-05

**Authors:** Peter C. Jenkins, Amber Lin, Stefanie G. Ames, Craig D. Newgard, Benjamin Lang, James E. Winslow, Jennifer R. Marin, Jennifer N. B. Cook, Jeremy D. Goldhaber-Fiebert, Linda Papa, Mark R. Zonfrillo, Matthew Hansen, Stephen P. Wall, Susan Malveau, Nathan Kuppermann

**Affiliations:** 1 Department of Surgery, Indiana University School of Medicine, Indianapolis; 2 Department of Emergency Medicine, Center for Policy and Research in Emergency Medicine, Oregon Health & Science University, Portland; 3 Department of Pediatrics, University of Utah School of Medicine, Salt Lake City; 4 Department of Pediatrics, Dell Medical School, University of Texas at Austin; 5 Department of Surgery, Dell Medical School, University of Texas at Austin; 6 Department of Emergency Medicine, Wake Forest School of Medicine, Winston-Salem, North Carolina; 7 North Carolina Office of Emergency Medical Services, Raleigh; 8 Departments of Pediatrics, University of Pittsburgh School of Medicine, Pittsburgh, Pennsylvania; 9 Department of Emergency Medicine, University of Pittsburgh School of Medicine, Pittsburgh, Pennsylvania; 10 Department of Radiology, University of Pittsburgh School of Medicine, Pittsburgh, Pennsylvania; 11 Centers for Health Policy, Primary Care, and Outcomes Research, Department of Medicine, Stanford University School of Medicine, Palo Alto, California; 12 Department of Emergency Medicine, Orlando Regional Medical Center, Orlando, Florida; 13 Department of Emergency Medicine, Alpert Medical School of Brown University, Providence, Rhode Island; 14 Department of Pediatrics, Alpert Medical School of Brown University, Providence, Rhode Island; 15 Ronald O. Perelman Department of Emergency Medicine, Department of Population Health, New York University School of Medicine, New York, New York; 16 Department of Emergency Medicine, University of California, Davis, School of Medicine, Sacramento

## Abstract

**Importance:**

Presentation to emergency departments (EDs) with high levels of pediatric readiness is associated with improved pediatric survival. However, it is unclear whether children of all races and ethnicities benefit equitably from increased levels of such readiness.

**Objective:**

To evaluate the association of ED pediatric readiness with in-hospital mortality among children of different races and ethnicities with traumatic injuries or acute medical emergencies.

**Design, Setting, and Participants:**

This cohort study of children requiring emergency care in 586 EDs across 11 states was conducted from January 1, 2012, through December 31, 2017. Eligible participants included children younger than 18 years who were hospitalized for an acute medical emergency or traumatic injury. Data analysis was conducted between November 2022 and April 2023.

**Exposure:**

Hospitalization for acute medical emergency or traumatic injury.

**Main Outcomes and Measures:**

The primary outcome was in-hospital mortality. ED pediatric readiness was measured through the weighted Pediatric Readiness Score (wPRS) from the 2013 National Pediatric Readiness Project assessment and categorized by quartile. Multivariable, hierarchical, mixed-effects logistic regression was used to evaluate the association of race and ethnicity with in-hospital mortality.

**Results:**

The cohort included 633 536 children (median [IQR] age 4 [0-12] years]). There were 557 537 children (98 504 Black [17.7%], 167 838 Hispanic [30.1%], 311 157 White [55.8%], and 147 876 children of other races or ethnicities [26.5%]) who were hospitalized for acute medical emergencies, of whom 5158 (0.9%) died; 75 999 children (12 727 Black [16.7%], 21 604 Hispanic [28.4%], 44 203 White [58.2%]; and 21 609 of other races and ethnicities [27.7%]) were hospitalized for traumatic injuries, of whom 1339 (1.8%) died. Adjusted mortality of Black children with acute medical emergencies was significantly greater than that of Hispanic children, White children, and of children of other races and ethnicities (odds ratio [OR], 1.69; 95% CI, 1.59-1.79) across all quartile levels of ED pediatric readiness; but there were no racial or ethnic disparities in mortality when comparing Black children with traumatic injuries with Hispanic children, White children, and children of other races and ethnicities with traumatic injuries (OR 1.01; 95% CI, 0.89-1.15). When compared with hospitals in the lowest quartile of ED pediatric readiness, children who were treated at hospitals in the highest quartile had significantly lower mortality in both the acute medical emergency cohort (OR 0.24; 95% CI, 0.16-0.36) and traumatic injury cohort (OR, 0.39; 95% CI, 0.25-0.61). The greatest survival advantage associated with high pediatric readiness was experienced for Black children in the acute medical emergency cohort.

**Conclusions and Relevance:**

In this study, racial and ethnic disparities in mortality existed among children treated for acute medical emergencies but not traumatic injuries. Increased ED pediatric readiness was associated with reduced disparities; it was estimated that increasing the ED pediatric readiness levels of hospitals in the 3 lowest quartiles would result in an estimated 3-fold reduction in disparity for pediatric mortality. However, increased pediatric readiness did not eliminate disparities, indicating that organizations and initiatives dedicated to increasing ED pediatric readiness should consider formal integration of health equity into efforts to improve pediatric emergency care.

## Key Points

**Question:**

Is increased emergency department (ED) pediatric readiness associated with an equitable decrease in the mortality of children of all races and ethnicities?

**Findings:**

In this cohort study of 633 536 children treated in 586 EDs across 11 states, mortality of Black children was greater than that of White children at all quartile levels of readiness among those with acute medical emergencies but not traumatic injuries. Increased readiness was associated with decreased mortality overall, and it decreased most for Black children with acute medical emergencies.

**Meaning:**

These findings suggest that increased ED pediatric readiness may reduce but not eliminate disparities among children with acute medical emergencies, indicating that organizations and initiatives dedicated to increasing ED pediatric readiness should consider formal integration of health equity into efforts to improve pediatric emergency care.

## Teaser

This cohort study examines emergency department (ED) pediatric readiness and differences in mortality among children of different races and ethnicities.

## Introduction

In response to large variations in the quality of pediatric emergency care found across US hospitals,^1^ the federal Emergency Medical Services for Children program established the National Pediatric Readiness Project (NPRP) in 2012. This national quality improvement initiative aims to ensure all emergency departments (EDs) have the necessary resources to adequately care for children with acute medical emergencies and traumatic injuries.^2^ Evidence that increased ED pediatric readiness is associated with improved survival in children with both acute medical emergencies and traumatic injuries underscores its importance.^3,4,5^ Accordingly, the American College of Surgeons incorporated an assessment of pediatric readiness into its 2022 criteria for trauma center verification.^6^ Although the benefits of increased ED pediatric readiness have been previously reported, it is unclear whether children of all races and ethnicities benefit equitably from such readiness.

Substantial evidence suggests that disparities exist in the treatment and outcomes of children according to race and ethnicity.^7,8,9,10,11,12,13,14,15,16,17^ Treatment protocols may be associated with reduced race and ethnicity disparities, presumably by mitigating the effects of biases.^18^ Because treatment protocols are an essential component of ED pediatric readiness, increased readiness may reduce such disparities through standardization of care.^19^ Moreover, increasing such readiness may be associated with reduced disparities more among children with acute medical emergencies compared with those with traumatic injuries, because treatment protocols for trauma are relatively well established.^6,20^ Therefore, although the initial purpose of the NPRP was to improve health care quality for children, the mission to increase ED pediatric readiness may also serve to promote health equity. The purpose of this study was to examine the association of ED pediatric readiness with racial and ethnic disparities in mortality among children with acute medical emergencies and traumatic injuries who presented to EDs in US states.

## Methods

### Study Design

We performed a secondary analysis of a retrospective cohort study of children receiving ED care in 11 states from January 1, 2012, through December 31, 2017. The study follows the Strengthening the Reporting of Observational Studies in Epidemiology (STROBE) reporting guideline and was approved by the institutional review boards at Oregon Health & Science University and the University of Utah, which waived the requirement for informed consent because the analysis was based on existing data and obtaining consent was not possible.

### Study Setting

We included 586 EDs with a matched NPRP assessment that cared for at least 10 children with acute medical emergencies and 10 children with traumatic injuries requiring hospitalization over the 6-year study period. The 11 states were Arizona, California, Florida, Iowa, Maryland, Minnesota, New Jersey, New York, North Carolina, Rhode Island, and Wisconsin. We selected geographically diverse states according to the availability of hospital and patient identifiers required to match the necessary data sources (described later) and included all states that granted approval to provide data.

### Patient Population

Eligible patients included all children younger than 18 years presenting to an ED who were hospitalized, defined as admission through the ED, transfer from the ED to another hospital, or death in the ED (January 1, 2012, through December 31, 2017). We linked visits chronologically at the patient level and designated the first ED visit as the index visit. Patients were excluded if they were missing the hospital disposition *discharged alive from the ED*, missing race or ethnicity data, or were treated at EDs without a matching NPRP assessment or EDs with fewer than 10 pediatric hospitalizations over the 6-year period. All analyses were performed separately for children with acute medical emergencies (ie, the medical cohort) and those with traumatic injuries (ie, the injury cohort).

### National Pediatric Readiness Assessment

The National Emergency Medical Services for Children Data Coordination Center conducted a national pediatric readiness assessment^2^ at 4149 of 5017 EDs (82.7%) in 50 states and US territories (2012-2013) based on national ED pediatric guidelines.^21^ The measure of ED pediatric readiness, called the weighted Pediatric Readiness Score, was developed by a national committee and 4 national emergency care and pediatric organizations from questions in 6 domains.^22^ The score ranges from 0 to 100, with 100 representing the maximal level of ED readiness (score 0-58, first quartile; score 59-72, second quartile; score 73-87, third quartile; and score 88-100, fourth quartile).^22^ The weighting schema was created by an expert panel and was used to quantify ED pediatric readiness across US hospitals^22^ and trauma centers.^5^ We matched the readiness data to the initial ED using hospital name, address, and zip code.

### Variables

We used race and ethnicity as recorded in the patient’s electronic medical record as part of standard state data collection practices. For the primary analysis, we excluded patients with missing race and ethnicity data. Due to the small sample sizes of some race and ethnicity categories, race and ethnicity were analyzed as non-Hispanic Black, Hispanic children of all races, non-Hispanic White, and non-Hispanic other (which included Asian, Hawaiian, Other Pacific Islander; American Indian or Alaska Native; multiple races; and any other race not specified). We refer to these cohorts as Black, Hispanic, other race, and White. Of the 11 states, 3 specified that race and ethnicity were self-reported, and others did not specify how race and ethnicity were recorded in the medical record. Other patient-level variables included demographics (age and sex), health insurance status (private, public, self-pay, or other), comorbidities,^23^ clinical severity assessed using the severity classification system^24^ (ranging from 1 to 5, with 5 being most severe), mechanism of injury (for patients with traumatic injuries only),^24,25^ Injury Severity Score (for patients with traumatic injuries only),^25,26^ surgical procedures, blood transfusion, interhospital transfer, length of hospital stay, and in-hospital mortality. To acquire information regarding ED and hospital procedures, we used the *International Classification of Diseases, Ninth Revision (ICD-9) *and *International Statistical Classification of Diseases and Related Health Problems, Tenth Revision (ICD-10)* procedure codes, categorized using the clinical classification system.^27^ We then mapped the clinical classification system categories to standardized operative domains and blood transfusion.^28^

### Statistical Analysis

We implemented multiple imputation separately for the injury cohort and medical cohort to address potential bias caused by missing data.^29^ We used the Stata mi-impute chained command, which uses flexible changed equations (Stata statistical software version 17, StataCorp).^30,31^ Our group has previously validated the use of multiple imputation in ED-based cohorts.^32,33^ Given that race and ethnicity (along with the weighted Pediatric Readiness Score) were primary exposure variables, we did not impute them for the primary analysis; however, we imputed race and ethnicity in a subsequent sensitivity analysis (discussed later). Descriptive statistics on missing data are shown in eTable 1 in Supplement 1.

To evaluate the association of race and ethnicity with mortality, we conducted a multivariable, mixed-effects logistic regression using the outcome of in-hospital mortality. The model included patient characteristics, illness, and injury characteristics as fixed effects and a random effect for facility to control for clustering.^34,35^ For race and ethnicity, we used the grand mean as the reference group. We based our models on a standardized risk-adjustment model for trauma that has been applied to children^3,4,36^ and modified for children with acute medical illness.^37^

Given previous findings of a mortality benefit associated with higher levels of ED pediatric readiness and the substantial evidence of racial disparities in pediatric emergency care,^4,8,14,15^ we sought to assess the association of ED pediatric readiness with in-hospital mortality among patients of different races and ethnicities. We first tested the association by including an interaction term between race and ethnicity and ED pediatric readiness quartile. In the models with the interaction term, we used White children as the reference group. We calculated the estimated probability of risk-adjusted mortality by race and ethnicity and quartile of ED pediatric readiness on the basis of these models and conducted pairwise comparisons between differences in race and ethnicity within each quartile of ED pediatric readiness. We implemented the Tukey-Kramer method to control for multiple testing.^38^ Multivariable modeling was conducted via the Stata xtmelogit command in combination with the Stata mi-estimate command to combine estimates across all imputations. Adjusted estimated probabilities were calculated using posterior prediction models with the Stata mimrgns command.^39^

To estimate the potential association of increasing ED pediatric readiness with racial disparities in mortality, we first calculated the difference between the point estimates of the estimated mortality of Black and White children in each quartile. These differences represent the racial disparities within each quartile. We then divided the mean disparity value of the lowest 3 quartiles by the disparity value of the fourth (highest readiness) quartile to yield an estimate of the association.

To examine the association of ED pediatric readiness with race and ethnicity, we conducted a number of a priori subgroup and sensitivity analyses of high-risk subgroups: those with high acuity (severity classification system score of ≥4), severe injuries (Injury Severity Score ≥16), sepsis (see eTable 2 in Supplement 1 for definition), respiratory distress (see eTable 2 in Supplement 1 for definition), and 1 or more comorbid conditions. We conducted 2 sensitivity analyses to determine the robustness of our primary analysis in handling of unknown and missing race and ethnicity data. The first analysis included patients with unknown race and ethnicity data as a missing indicator, and the second analysis used imputed race and ethnicity. We also conducted another sensitivity analysis to determine whether facility characteristics (trauma level, pediatric ED, hospital type, and patient diversity) changed the results of the primary analysis. To assess diversity as a hospital characteristic, we calculated the percentage of patients of a race or ethnicity other than White admitted at each hospital and divided them into quartiles. All statistical tests were 2-sided with an α of .05. All data analyses were conducted between November 2022 and April 2023.

## Results

The cohort included a total of 633 536 children (median [IQR] age, 4 [0-12] years) including 557 537 children with acute medical emergencies and 75 999 with traumatic injuries (eFigure in Supplement 1). The medical cohort consisted of 98 504 Black children (17.7%), 167 838 Hispanic children (30.1%), 311 157 White children (55.8%), and 147 876 children of other races (26.5%). The injury cohort consisted of 12 727 Black children (16.7%), 21 604 Hispanic children (28.4%), 44 203 White children (58.2%), and 21 609 children of other races (27.7%). Table 1 describes the sample stratified by acute medical emergencies and traumatic injuries, and eTable 3 in Supplement 1 details the distribution of patients by race and ethnicity and quartile of ED pediatric readiness.

**Table 1.** Summary of Patient Characteristics

Characteristic	Patients, No. (%) (N = 633 536)	
	Acute medical emergencies (n = 557 537)	Traumatic injuries (n = 75 999)
Age, y		
<1	196 783 (35.3)	6695 (8.8)
1-4	112 558 (20.2)	14 260 (18.8)
5-9	82 119 (14.7)	16 273 (21.4)
10-12	48 368 (8.7)	9500 (12.5)
13-15	63 928 (11.5)	15 074 (19.8)
16-17	53 781 (9.6)	14 197 (18.7)
Sex		
Female	258 140 (46.3)	27 732 (36.5)
Male	299 397 (53.7)	48 267 (63.5)
Race		
Black	98 504 (17.7)	12 727 (16.7)
White	311 157 (55.8)	44 203 (58.2)
Other^a^	147 876 (26.5)	19 069 (25.1)
Hispanic ethnicity	167 838 (30.1)	21 604 (28.4)
Comorbidities		
None	476 576 (85.5)	70 640 (92.9)
1	55 287 (9.9)	4055 (5.3)
≥2	25 674 (4.6)	1304 (1.7)
Health insurance type		
Private	235 903 (42.3)	33 961 (44.7)
Public	286 544 (51.4)	31 802 (41.8)
Self-pay	26 124 (4.7)	4941 (6.5)
Other	8966 (1.6)	5295 (7.0)
Mechanism of injury		
Firearm	NA	1983 (2.6)
Stab or penetrating	NA	3875 (5.1)
Assault	NA	7221 (9.5)
Fall	NA	28 294 (37.2)
Motor vehicle	NA	9586 (12.6)
Pedestrian or bicycle	NA	7805 (10.3)
Other	NA	17 235 (22.7)
Injury Severity Score		
0-8	NA	52 737 (69.4)
9-15	NA	18 021 (23.7)
16-24	NA	2981 (3.9)
≥25	NA	2260 (3.0)
Severity classification score ≥4	294 740 (52.9)	37 922 (49.9)
Blood transfusion within 24 h	4922 (0.9)	1239 (1.6)
Major surgery	73 026 (13.1)	6102 (8.0)
Orthopedic surgery	3393 (0.6)	28 868 (38.0)
Interhospital transfer	30 761 (5.5)	5666 (7.5)
In-hospital mortality	5158 (0.9)	1339 (1.8)

Abbreviation: NA, not applicable.

^a^ Other was defined as American Indian and Alaska Native; Asian, Hawaiian, and Other Pacific Islander; multiple races; and any other race not specified.

After controlling for patient and illness characteristics, the mortality of Black patients was significantly greater than that of Hispanic patients, White patients, and patients of other races in the medical cohort (odds ratio [OR], 1.69; 95% CI, 1.59-1.79) but was no different in the injury cohort (OR, 1.01; 95% CI, 0.89-1.15). In both the medical and injury cohorts, Hispanic ethnicity was associated with decreased mortality compared with patients who were non-Hispanic. Table 2 describes the association of each variable in the adjusted model with mortality. At hospitals with the highest ED pediatric readiness scores (fourth quartile), mortality was significantly lower for children of all races and ethnicities in both the medical cohort (OR, 0.24; 95% CI, 0.16-0.36) and the injury cohort (OR, 0.39; 95% CI, 0.25-0.61) compared with hospitals with the lowest ED pediatric readiness scores (first quartile). However, across all quartiles of the ED pediatric readiness score, Black children with acute medical emergencies had greater estimated probability of in-hospital mortality than other children (Figure). The C statistic was 0.91 for the medical cohort and 0.94 for the injury cohort.

**Table 2.** Multivariable Models of Mortality by Patient Characteristics and ED Pediatric Readiness

Characteristic	OR (95% CI)	
	Patients with acute medical emergencies (n = 557 537)	Patients with traumatic injuries (n = 75 999)
ED weighted Pediatric Readiness Score quartile		
1 (0-58)	1 [Reference]	1 [Reference]
2 (59-72)	0.88 (0.57-1.35)	1.08 (0.66-1.74)
3 (73-87)	0.68 (0.44-1.03)	0.99 (0.62-1.58)
4 (88-100)	0.24 (0.16-0.36)	0.39 (0.25-0.61)
Race and ethnicity^a^		
Black	1.69 (1.59-1.79)	1.01 (0.89-1.15)
Hispanic	0.57 (0.53-0.60)	0.88 (0.78-0.99)
Other^b^	1.02 (0.95-1.09)	1.10 (0.95-1.28)
White	1.03 (0.98-1.09)	1.02 (0.92-1.14)
Sex		
Female	0.88 (0.82-0.93)	1.04 (0.90-1.19)
Male	1 [Reference]	1 [Reference]
Age group, y		
0	1 [Reference]	1 [Reference]
1-4	0.48 (0.44-0.53)	0.98 (0.77-1.24)
5-9	0.23 (0.20-0.25)	0.54 (0.42-0.71)
10-12	0.22 (0.19-0.25)	0.43 (0.32-0.57)
13-15	0.16 (0.14-0.18)	0.28 (0.21-0.36)
16-17	0.1 (0.09-0.12)	0.24 (0.18-0.31)
Health insurance type		
Public	1 [Reference]	1 [Reference]
Private	0.85 (0.78-0.92)	1.04 (0.85-1.26)
Self-pay	4.72 (4.26-5.23)	5.07 (4.05-6.35)
Other	1.43 (1.11-1.86)	0.94 (0.71-1.23)
Comorbidities		
None	1 [Reference]	1 [Reference]
1	1.36 (1.23-1.49)	2.35 (1.96-2.83)
≥2	3.25 (2.93-3.61)	3.55 (2.77-4.54)
Transfer out	0.03 (0.02-0.03)	0.23 (0.16-0.31)
Severity classification score		
1-3	1 [Reference]	1 [Reference]
4-5	66.46 (51.09-86.44)	5.04 (3.86-6.57)
Blood transfusion ≤24 h	3.40 (2.80-4.13)	2.12 (1.71-2.64)
Injury Severity Score		
0-8	NA	1 [Reference]
9-15	NA	4.4 (3.50-5.54)
16-24	NA	13.28 (10.31-17.10)
≥25	NA	19.1 (14.83-24.61)
Mechanism of injury		
Fall	NA	1 [Reference]
Firearm	NA	37.32 (26.71-52.15)
Stabbing or penetrating injury	NA	8.98 (5.83-13.82)
Assault	NA	3.55 (2.42-5.20)
Motor vehicle	NA	5.95 (4.39-8.06)
Bicycle or pedestrian	NA	8.12 (5.99-11.01)
Other	NA	4.79 (3.60-6.36)

Abbreviations: ED, emergency department; OR, odds ratio.

^a^ The reference for race and ethnicity is the grand mean.

^b^ Other was defined as American Indian and Alaska Native; Asian, Hawaiian, and Other Pacific Islander; multiple races; and any other race not specified.

**Figure.**
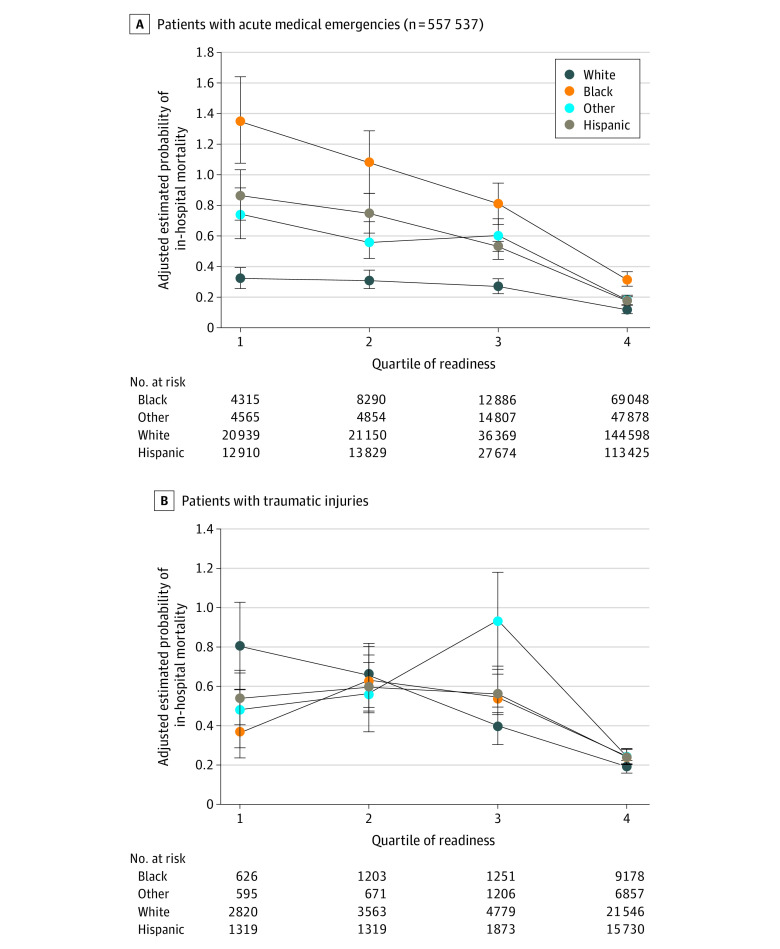
Risk-Adjusted Mortality by Quartile of the Weighted Pediatric Readiness Score and Race and Ethnicity The figure shows the adjusted estimated probabilities of in-hospital pediatric mortality for patients with acute medical emergencies (A) and traumatic injuries (B) stratified by race and ethnicity and by emergency department readiness quartile (weighted pediatric readiness score 0-58, first quartile; score 59-72, second quartile; score 73-87, third quartile; and score 88-100 fourth quartile). Dots denote means and error bars denote 95% CIs. Other was defined as American Indian and Alaska Native; Asian, Hawaiian, and Other Pacific Islander; and multiple races.

In the medical cohort, although the mortality of Black children was greater than that of Hispanic children, children of other races or ethnicities, and White children, the difference incrementally decreased as levels of ED pediatric readiness increased (Figure), and the association of ED pediatric readiness with race and ethnicity was statistically significant (*P* for interaction = .005). Among injured children, there were no significant associations of race with mortality (Table 2), although mortality associated with Hispanic ethnicity was significantly lower than that for the reference group (OR, 0.88; 95% CI, 0.78-0.99). In the injury cohort, there was no significant difference for race and ethnicity in the estimated probability of mortality across levels of ED pediatric readiness, although Hispanic mortality decreased most consistently (Figure). Accordingly, the association of ED pediatric readiness with race and ethnicity in the injury cohort was not significant (*P* for interaction = .11).

In the cohort of children with acute medical emergencies, the estimated racial disparity in hospitals with the highest quartile of ED pediatric readiness was 0.14%, and the mean disparity of the hospitals in the lowest 3 quartiles of ED pediatric readiness was 0.49%. Therefore, we estimated that the racial disparity in hospitals with the highest ED pediatric readiness scores was 3-fold less than the hospitals in the lowest 3 quartiles. We did not perform a similar estimate for the injury cohort, because the association of race with mortality was not statistically significant.

All sensitivity and subgroup analyses were consistent with the primary results, with 2 exceptions. For all subgroup analyses within the medical cohort, Black race was consistently associated with significantly higher odds of mortality, and Hispanic ethnicity was associated with decreased odds of mortality. Also, among those in the medical cohort with 1 or more comorbid conditions, both Black race (OR, 1.28; 95% CI, 1.14-1.44) and other race (OR, 1.15; 95% CI, 1.01-1.31) were associated with significantly increased odds of mortality compared with the reference group.). Results of the sensitivity analysis are summarized in eTable 4 in Supplement 1.

The findings from our primary analysis were robust for different methods of addressing missing data (eTable 2 in Supplement 1). When missing race and ethnicity data were imputed or included in the analyses as a missing indicator, results regarding race and ethnicity were similar to those for the primary analysis. When unknown race and ethnicity were included in the analysis, it was significantly associated with increased odds of mortality compared with all other patients.

When evaluating the associations of hospital factors, we found the primary results were robust for these tests. However, certain hospital factors (trauma levels of I and II, pediatric ED status, and children’s hospitals) were all associated with decreased mortality compared with their counterparts. Most notably, the least ethnically and racially diverse hospitals had significantly greater mortality than more diverse hospitals (eTable 5 in Supplement 1).

## Discussion

In this cohort study, we examined the association of ED pediatric readiness with race and ethnicity disparities in mortality among 2 large cohorts of children receiving emergency services. Among children who presented with acute medical emergencies, Black children were more likely to die than White children across all levels of ED pediatric readiness, but there were no such mortality disparities among children with traumatic injuries. In the medical cohort, the mortality of Black children decreased more than that of children of other races and ethnicities in EDs with higher levels of readiness. As a result, the racial disparity in mortality narrowed with increased ED pediatric readiness but was not eliminated, even at the highest pediatric readiness level.

These results have notable policy implications for programs dedicated to increasing ED pediatric readiness. Thus far, such programs have largely focused on the survival benefit associated with ED pediatric readiness. Our results suggest that ED pediatric readiness may serve to promote health equity among children as well. Despite this potential benefit, disparities existed across all quartile levels of readiness among children with acute medical emergencies. This finding represents an opportunity for initiatives such as the NPRP to formally incorporate health equity into their national platform and engage with the issue in a structured, systematic manner. With incorporation of health equity into such initiatives, organizations may begin to address racial disparities both as a moral imperative as well as a measure of health care quality.

Conceptual frameworks currently exist that outline how to evaluate racial and ethnic health equity as a quality metric.^40,41^ Programs focused on ED pediatric readiness are ideally positioned to adopt such a framework because ED pediatric readiness consists of well-defined, largely modifiable factors.^20^ The NPRP administered an updated national assessment of ED readiness in 2021, which may offer an opportunity to evaluate the association of the domains of ED pediatric readiness with racial disparities in more depth when these data become available. Such work may clarify which aspects of ED pediatric readiness contribute most to health equity, so organizations can incorporate a health equity framework to improve the quality and consistency of pediatric emergency care in US hospitals.

The lack of racial and ethnic disparities in mortality in the cohort of children with traumatic injuries has notable policy implications. As stated previously, trauma care is highly protocolized, and these findings support the notion that such structured systems of care promote racial and ethnic equity in pediatric outcomes. However, another explanation for the finding that disparities existed in the medical cohort but not in the injury cohort is that the injury cohort was a healthier patient population. Because the burden of comorbidities among children in the medical cohort was substantially greater than in the injury cohort, those preexisting comorbidities, which are often the result of experienced racism and structural inequities, may have predisposed Black children to greater mortality than White children in the medical cohort. Also, because the injury cohort consisted of a smaller sample size than the medical cohort, this study may have been underpowered to detect a difference between race categories.

Notably, Hispanic ethnicity was associated with decreased risk of mortality in both cohorts. Particularly in the injury cohort, mortality of Hispanic children decreased more consistently than for children of other races and ethnicities as ED pediatric readiness increased. Literature regarding risk of mortality associated with Hispanic ethnicity has had mixed results,^8,42,43,44^ and we are wary of overinterpreting how biases and racism impacted the mortality findings among Hispanic children in both cohorts.

The examination of hospital characteristics found that hospitals with the least amount of racial and ethnic diversity experienced the greatest mortality. This finding—that segregation at the facility-level is associated with clinical outcomes—is consistent with prior studies.^45,46,47^ However, it has not been previously reported in the pediatric population. This finding clearly warrants further investigation.

### Limitations

The results of this study should be interpreted within the context of its limitations. The recording of race and ethnicity was performed in a heterogeneous manner. Although certain states used patient-identified race and ethnicity, others relied on clinicians to identify patient race and ethnicity (or did not specify how race and ethnicity data were collected). Unfortunately, this inconsistency is inherent to administrative data collected at the state level across hundreds of hospitals in multiple states. Also, due to small sample sizes, we were unable to disaggregate certain racial categories. Specifically, we had to combine Asian, Hawaiian, and Other Pacific Islander; American Indian, and Alaska Native; multiple races, and any other race not specified into a single category (other) for stability and convergence of the statistical models. Collapsing these categories may have obscured important and varying associations with the study outcome. Another potential limitation of the study is that the documentation of race and ethnicity as missing and unknown may have occurred in a nonrandom manner and introduced unmeasured biases. Missing and unknown race and ethnicity were associated with increased mortality, perhaps due to incomplete documentation during and after life-threatening emergencies (eg, children who died early had a higher proportion of missing race and ethnicity data). To address these biases, we performed multiple sensitivity analyses, and although the results generally supported our primary findings, the data were inherently limited. Furthermore, we used ED pediatric readiness data collected in 2013, and it is possible that the readiness of individual EDs has changed over time.

## Conclusions

In this cohort study of 633 536 children, racial and ethnic disparities in mortality existed among children treated for acute medical emergencies but not traumatic injuries. Increased ED pediatric readiness was associated with reduced disparities in mortality but did not eliminate them. Organizations and initiatives dedicated to increasing ED pediatric readiness should consider formal integration of health equity into efforts to improve pediatric emergency care.

## Notes

Supplement 1. **eTable 1.** Summary of Missing Data **eTable 2.** Sensitivity Analyses Addressing Missing Data **eFigure.** CONSORT Diagram **eTable 3.** Distribution of Race and Ethnicity by Quartile of ED Pediatric Readiness **eTable 4.** Sensitivity Analyses of Subgroups **eTable 5.** Sensitivity Analyses Addressing Hospital Characteristics

Supplement 2.
Nonauthor Collaborators

Supplement 3.
Data Sharing Statement
