# Supplementary material for: Emergency Department Pediatric Readiness and Disparities in Mortality Based on Race and Ethnicity
Source: JAMA Netw Open. 2023 Sep 5;6(9):e2332160. doi: 10.1001/jamanetworkopen.2023.32160 (PMC10481245; doi:10.1001/jamanetworkopen.2023.32160)
Supplement: Supplement 1. — eTable 1. Summary of Missing Data eTable 2. Sensitivity Analyses Addressing Missing Data eFigure. CONSORT Diagram eTable 3. Distribution of Race and Ethnicity by Quartile of ED Pediatric Readiness eTable 4. Sensitivity Analyses of Subgroups eTable 5. Sensitivity Analyses Addressing Hospital Characteristics [file jamanetwopen-e2332160-s001.pdf]

Supplemental Online Content

Jenkins PC, Lin A, Ames SG, et al; Pediatric Readiness Group. Emergency department pediatric readiness and disparities in mortality based on race and ethnicity. *JAMA Netw Open*. 2023; 6(9):e2332160 doi:10.1001/jamanetworkopen.2023.32160

eTable 1. Summary of Missing Data
eTable 2. Sensitivity Analyses Addressing Missing Data
eFigure. CONSORT Diagram
eTable 3. Distribution of Race and Ethnicity by Quartile of ED Pediatric Readiness
eTable 4. Sensitivity Analyses of Subgroups
eTable 5. Sensitivity Analyses Addressing Hospital Characteristics

This supplemental material has been provided by the authors to give readers additional information about their work.

© 2023 Jenkins PC et al. *JAMA Network Open.*

eTable 1. Summary of Missing Data

**Patient, injury/illness characteristics**	**Medical Cohort n = 557,537 n(%)**	**Injury Cohort n = 75,999 n(%)**
Age	0 (0.0%)	0 (0.0%)
Female	29 (0.0%)	13 (0.0%)
Race*	0 (0.0%)	0 (0.0%)
Hispanic/Latino ethnicity*	0 (0.0%)	0 (0.0%)
Comorbidities	0 (0.0%)	0 (0.0%)
Payer	163,758 (29.4%)	26,131 (34.4%)
Mechanism of injury	NA	8,123 (10.7%)
Injury severity	NA	7,429 (9.8%)
Severity of illness	18,139(3.3%)	1,942 (2.6%)
Blood transfusion	0 (0.0%)	0 (0.0%)
Major surgery	0 (0.0%)	0 (0.0%)
Orthopedic surgery	0 (0.0%)	0 (0.0%)
Inter-hospital transfer	0 (0.0%)	0 (0.0%)
In-hospital mortality	0 (0.0%)	0 (0.0%)

*Patients missing race and ethnicity were excluded in the last step of the inclusion/exclusion criteria. Prior to exclusion of missing values, race was missing in 80,806(11.8%) of medically ill and 9,553 (10.5%) of injured patients; ethnicity was missing in 96,058(14.1%) of medically ill and 11,793(13.0%) of injured patients; and both race and ethnicity were missing in 51,398(7.5%) of medical ill and 6,439(7.1%) of injured patients.

© 2023 Jenkins PC et al. *JAMA Network Open.*

eTable 2. Distribution of Race and Ethnicity by Quartile of ED Pediatric Readiness*

	Medical Cohort (n = 602,197)			
	**1st quartile** n=42,729 (Least ready)	**2nd quartile** n=48,123	**3rd quartile** n=91,736	**4th quartile** n=374,949 (Most ready)
**Race/Ethnicity, n(%)**				
Black	4441(10.4%)	8621(17.9%)	13282(14.5%)	72160(19.3%)
Asian/ Hawaiian/ Pacific Islander	2393(5.6%)	2452(5.1%)	6575(7.2%)	19479(5.2%)
American Indian/ Alaska Native	517(1.2%)	956(2%)	686(0.8%)	3002(0.8%)
White	28257(66.1%)	28413(59%)	51377(56%)	203110(54.2%)
Other/Multiple	7121(16.7%)	7681(16%)	19816(21.6%)	77198(20.6%)
Hispanic	12910(30.2%)	13829(28.7%)	27674(30.2%)	113425(30.3%)
	**Injury Cohort (n = 81,353)**			
	**1st quartile** n=5,360 (Least ready)	**2nd quartile** n=7,310	**3rd quartile** n=10,018	**4th quartile** n=53,311 (Most ready)
**Race/Ethnicity, n(%)**				
Black	640(11.9%)	1252(17.1%)	1283(12.8%)	9552(17.9%)
Asian/ Hawaiian/ Pacific Islander	247(4.6%)	346(4.7%)	591(5.9%)	2443(4.6%)
American Indian/ Alaska Native	99(1.9%)	81(1.1%)	80(0.8%)	545(1%)
White	3575(66.7%)	4309(59%)	6403(63.9%)	29916(56.1%)
Other/Multiple	799(14.9%)	1322(18.1%)	1661(16.6%)	10855(20.4%)
Hispanic	1319(24.6%)	1873(25.6%)	2682(26.8%)	15730(29.5%)

© 2023 Jenkins PC et al. *JAMA Network Open.*

eFigure. CONSORT Diagram

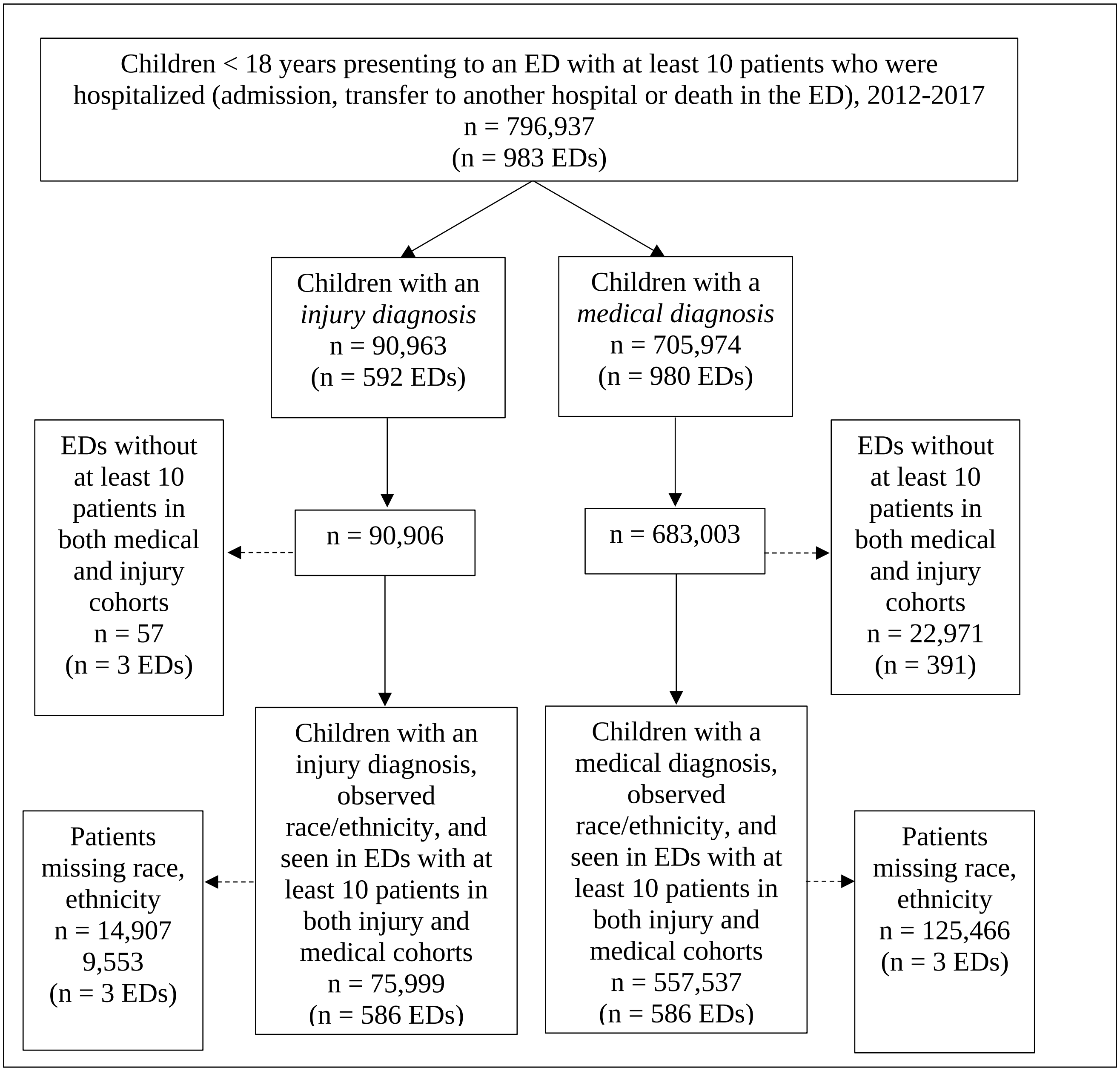

© 2023 Jenkins PC et al. *JAMA Network Open.*

eTable 3. Sensitivity Analyses of Subgroups

	Injured children	Medical children
**Primary Analysis**	OR (95% CI)	OR (95% CI)
Race/Ethnicity (reference is grand mean)	(n = 75,999)	(n = 557,537)
White	1.02(0.92-1.14)	1.03(0.98-1.09)
Black	1.01(0.89-1.15)	1.69(1.59-1.79)
Other	1.1(0.95-1.28)	1.02(0.95-1.09)
Hispanic	0.88(0.78-0.99)	0.57(0.53-0.6)
**Severely Ill (Severity Classification Score ≥4)**		
Race/Ethnicity (reference is grand mean)	(n = 37,046)	(n =287,627)
White	1.03(0.91-1.16)	1.01(0.96-1.07)
Black	1.02(0.89-1.17)	1.68(1.59-1.79)
Other	1.11(0.94-1.3)	1.03(0.96-1.11)
Hispanic	0.87(0.76-0.99)	0.57(0.53-0.61)
**Severely Injured (Injury Severity Score ≥16)**	(n =5,000)	
Race/Ethnicity (reference is grand mean)		
White	1.08(0.9-1.3)	NA
Black	0.98(0.79-1.23)	NA
Other	1.09(0.83-1.43)	NA
Hispanic	0.86(0.7-1.06)	NA
**Subgroup of Respiratory Distress or Respiratory Failure**		
Race/Ethnicity (reference is grand mean)		(n = 19,397)
White	NA	1.01(0.89-1.15)
Black	NA	1.23(1.07-1.42)
Other	NA	1.15(0.98-1.37)
Hispanic	NA	0.7(0.6-0.82)
**Subgroup of Sepsis Diagnosis and Severity Score 4,5**		
Race/Ethnicity (reference is grand mean)		(n =48,741)
White	NA	0.97(0.84-1.13)
Black	NA	1.32(1.11-1.56)
Other	NA	1.04(0.86-1.26)
Hispanic	NA	0.77(0.64-0.92)
**One or more comorbid conditions**		
Race/Ethnicity (reference is grand mean)		(n = 80,961)
White	NA	0.84(0.76-0.94)
Black	NA	1.28(1.14-1.44)
Other	NA	1.15(1.01-1.31)
Hispanic	NA	0.8(0.71-0.9)

© 2023 Jenkins PC et al. *JAMA Network Open.*

eTable 4. Sensitivity Analyses Addressing Missing Data

	Injured children	Medical children
	OR (95% CI)	OR (95% CI)
**Primary Analysis**		
Race/Ethnicity (reference is grand mean)	(n = 75,999)	(n = 557,537)
White	1.02(0.92-1.14)	1.03(0.98-1.09)
Black	1.01(0.89-1.15)	1.69(1.59-1.79)
Other	1.1(0.95-1.28)	1.02(0.95-1.09)
Hispanic	0.88(0.78-0.99)	0.57(0.53-0.6)
**Unknown race and ethnicity included**	(n = 90,906)	(n = 683,003)
Race/Ethnicity (reference is grand mean)		
White	0.77(0.7-0.86)	0.82(0.77-0.86)
Black	0.76(0.67-0.86)	1.36(1.29-1.45)
Other	0.76(0.68-0.85)	0.84(0.78-0.9)
Hispanic	0.87(0.75-1.01)	0.48(0.45-0.51)
Unknown race and ethnicity versus else	2.57(2.14-3.1)	2.23(2.01-2.48)
**Race imputed**	(n = 90,906)	(n = 683,003)
Race/Ethnicity (reference is grand mean)		
White	1.01(0.92-1.11)	1.01(0.96-1.05)
Black	0.98(0.88-1.1)	1.71(1.62-1.8)
Other	1.11(0.98-1.27)	1.02(0.96-1.09)
Hispanic	0.9(0.82-1)	0.57(0.54-0.6)

© 2023 Jenkins PC et al. *JAMA Network Open.*

eTable 5. Sensitivity Analyses Addressing Hospital Characteristics

	**Injured children**	**Medical children**
**Primary Analysis**	OR (95% CI)	OR (95% CI)
Race/Ethnicity (reference is grand mean)	(n = 75,999)	(n = 557,537)
White	1.02(0.92-1.14)	1.03(0.98-1.09)
Black	1.01(0.89-1.15)	1.69(1.59-1.79)
Other	1.1(0.95-1.28)	1.02(0.95-1.09)
Hispanic	0.88(0.78-0.99)	0.57(0.53-0.6)
ED Pediatric Readiness Score		
1st quartile (wPRS 0-58)	referent	referent
2nd quartile (wPRS 59-72)	1.08(0.66-1.74)	0.88(0.57-1.35)
3rd quartile (wPRS 73-87)	0.99(0.62-1.58)	0.68(0.44-1.03)
4th quartile (wPRS 88-100)	0.39(0.25-0.61)	0.24(0.16-0.36)
**Adding highest TC level as a predictor**		
Race/Ethnicity (reference is grand mean)		
White	1.01(0.91-1.12)	1.03(0.98-1.08)
Black	1.03(0.9-1.17)	1.51(1.39-1.64)
Other	1.1(0.94-1.27)	1.02(0.95-1.1)
Hispanic	0.88(0.78-0.99)	0.64(0.55-0.73)
ED Pediatric Readiness Score		
1st quartile (wPRS 0-58)	referent	referent
2nd quartile (wPRS 59-72)	1.12(0.72-1.75)	0.88(0.57-1.34)
3rd quartile (wPRS 73-87)	1.07(0.7-1.64)	0.68(0.45-1.03)
4th quartile (wPRS 88-100)	0.61(0.4-0.93)	0.29(0.19-0.45)
Highest TC level		
Non-trauma center	referent	referent
Level 1 Adult or Pediatric	0.33(0.23-0.46)	0.56(0.36-0.86)
Level 2 Adult or Pediatric	0.53(0.38-0.73)	1.13(0.77-1.64)
Level 3 Adult or Pediatric	1.26(0.78-2.05)	1.62(1.02-2.58)
Level 4/5 Adult or Pediatric	1.72(0.52-5.76)	0.97(0.33-2.89)
**Adding pediatric ED as a predictor**		
Race/Ethnicity (reference is grand mean)		
White	1.01(0.91-1.12)	1.03(0.97-1.08)
Black	1.02(0.9-1.16)	1.7(1.6-1.8)
Other	1.1(0.95-1.28)	1.02(0.95-1.09)
Hispanic	0.88(0.78-0.99)	0.56(0.53-0.6)
ED Pediatric Readiness Score		
1st quartile (wPRS 0-58)	referent	referent
2nd quartile (wPRS 59-72)	1.09(0.68-1.74)	0.84(0.56-1.27)
3rd quartile (wPRS 73-87)	1.04(0.66-1.65)	0.74(0.5-1.11)
4th quartile (wPRS 88-100)	0.63(0.39-0.99)	0.44(0.29-0.67)
Pediatric ED status		
No	referent	referent
Yes	0.39(0.27-0.56)	0.23(0.16-0.34)

© 2023 Jenkins PC et al. *JAMA Network Open.*

**Adding hospital type**		
Race/Ethnicity (reference is grand mean)		
White	1.01(0.9-1.12)	1.03(0.97-1.08)
Black	1.02(0.9-1.16)	1.69(1.6-1.79)
Other	1.1(0.95-1.28)	1.02(0.95-1.09)
Hispanic	0.88(0.78-0.99)	0.57(0.53-0.6)
ED Pediatric Readiness Score		
1st quartile (wPRS 0-58)	referent	referent
2nd quartile (wPRS 59-72)	1.15(0.73-1.81)	0.89(0.59-1.36)
3rd quartile (wPRS 73-87)	1.09(0.7-1.69)	0.69(0.46-1.04)
4th quartile (wPRS 88-100)	0.55(0.36-0.85)	0.33(0.22-0.49)
Hospital type		
Non-children's, non-academic, non-Level I/II trauma center	referent	referent
Non-children's, non-university, academic affiliated	0.76(0.41-1.39)	0.56(0.31-1.02)
Non-children's, non-academic, Level II trauma center	0.6(0.35-1.04)	1.14(0.62-2.1)
Non-children's, ACGME-accredited academic hospitals	0.48(0.35-0.66)	0.58(0.43-0.78)
Children's hospital	0.3(0.19-0.5)	0.19(0.1-0.35)
**Adding quartile of non-White**		
Race/Ethnicity (reference is grand mean)		
White	1.02(0.91-1.13)	1.02(0.97-1.08)
Black	1.02(0.89-1.15)	1.7(1.6-1.8)
Other	1.1(0.95-1.28)	1.02(0.95-1.09)
Hispanic	0.88(0.78-0.99)	0.56(0.53-0.6)
ED Pediatric Readiness Score		
1st quartile (wPRS 0-58)	referent	referent
2nd quartile (wPRS 59-72)	1.08(0.66-1.75)	0.86(0.56-1.32)
3rd quartile (wPRS 73-87)	0.98(0.61-1.57)	0.67(0.44-1.02)
4th quartile (wPRS 88-100)	0.42(0.27-0.65)	0.26(0.17-0.4)
ED quartile of non-white pediatric patients		
<28.6% non-white	referent	referent
28.6 to <48.3% non-white	0.67(0.42-1.08)	0.54(0.35-0.83)
48.3% to <68.9% non-white	0.66(0.41-1.09)	0.5(0.32-0.79)
≥68.9% non-white	0.74(0.44-1.22)	0.51(0.32-0.82)

© 2023 Jenkins PC et al. *JAMA Network Open.*
