# Supplementary material for: Emergency Department Pediatric Readiness and Disparities in Mortality Based on Race and Ethnicity
Source: JAMA Netw Open. 2023 Sep 5;6(9):e2332160. doi: 10.1001/jamanetworkopen.2023.32160 (PMC10481245; doi:10.1001/jamanetworkopen.2023.32160)
Supplement: Supplement 2. — Nonauthor Collaborators [file jamanetwopen-e2332160-s002.pdf]

Supplemental Online Content: Nonauthor Collaborators
*First name, last name, and suffix (if applicable) are required and will appear in PubMed.

*Group Name: Pediatric Readiness Study Group							
***First Name and Middle Initial(s)**	***Last Name**	***Suffix (eg, Jr, III)**	Academic Degrees	Institution	Location (city, state/province, country)	Role or Contribution, eg, chair, principal investigator	Group (if more than 1 Group listed in the byline) and/or Subgroup (eg, Steering Committee)
Nina	Glass		MD	Rutgers University	Newark, New Jersey, USA	Collaborator	
Clay	Mann		PhD, MS	University of Utah	Salt Lake City, Utah, USA	Investigator	
Avery	Nathens		MD, MPH, PhD	Sunnybrook Research Institute	Toronto, Ontario, CA	Investigator	
John	McConnell		PhD	Oregon Health Sciences University	Portland, Oregon, USA	Investigator	
Marianne	Gausche		MD	David Geffen School of Medicine at UCLA and Los Angeles County Emergency Medical Services Agency	Los Angeles, CA, USA	Investigator	
Angela	Child		MS	University of Utah	Salt Lake City, Utah, USA	Study staff/analytic team	
Mengtao	Dai		MS	University of Utah	Salt Lake City, Utah, USA	Study staff/analytic team	
Apoorva	Salvi		MPH	Oregon Health Sciences University	Portland, Oregon, USA	Study staff/analytic team	
Brendan	Carr		MD, MS	Mount Sinai	New York, New York, USA	Advisory Council	
Kate	Remick		MD	University of Texas at Austin	Austin, Texas, USA	Advisory Council	
Ryan	Mutter		PhD	U.S. Congressional Budget Office	Washington, DC, USA	Advisory Council	
Hilary	Hughes		MD	University of Utah	Salt Lake City, Utah, USA	Advisory Council	
Randall	Burd		MD, PhD	Children's Ntl Medical Center	Washington, DC, USA	Advisory Council	
Rachel	Ford		MD	Children's Ntl Medical Center	Washington, DC, USA	Advisory Council	
