# Supplementary material for: Emergency Department Pediatric Readiness and Disparities in Mortality Based on Race and Ethnicity
Source: JAMA Netw Open. 2023 Sep 5;6(9):e2332160. doi: 10.1001/jamanetworkopen.2023.32160 (PMC10481245; doi:10.1001/jamanetworkopen.2023.32160)
Supplement: Supplement 3. — Data Sharing Statement [file jamanetwopen-e2332160-s003.pdf]

# Data Sharing Statement

Jenkins. Emergency Department Pediatric Readiness and Disparities in Mortality Based on Race and Ethnicity. *JAMA Netw Open*. Published September 05, 2023.
doi:10.1001/jamanetworkopen.2023.32160

**Data**
**Data available:** No

**Additional Information**
**Explanation for why data not available:** The restrictive nature of the data use agreements prevents us from being able to share data.
